# To the Editors of the Medical and Physical Journal

**Published:** 1800-11

**Authors:** Tuaam Peaal

**Affiliations:** Aberdeen


### *To the Editors of the Medical and Physical Journal.*

Gentlemen,

If the following Case and Observations upon Polydipsia are thought interesting to your readers, or suited to afford Dr. Dyce a few hints upon the treatment of this affection, they are at your service.

J. F. a man aged about 45 years, of a dark complexion, well boned and muscular, but not inclined to corpulency, has been affected for two months with violent thirst, which has never had any remission since he was first affected with it. In the night time, he says, that his thirst is very great, and that he puts down a large decanter, containing one Scots pint of water, at his bedside, which is not sufficient for him during the night. In the twenty-four hours he will drink more than two gallons. He says that he has no other complaint but thirst, except that he is generally costive. His appetite is much impaired, and he has seldom a desire for food; he sweats profusely during the night and towards morning, and is often obliged to change his linen. Sometimes the quantity of water which he drinks makes his belly feel full, hard, and painful. He sleeps pretty well some nights, but is often obliged to rise early in the morning, on account of the thirst and sweating. Since he has been affected with this complaint he is more dejected in his spirits than formerly, and has not the same desire for working; this, he says, is owing to his strength being somewhat diminished. He secretes a large quantity of urine, and voids it without pain; it has much the appearance of the urine of diabetic patients. His pulse is about 90 in the minute. Three years since he conducted a distillery of whisky, and was then liable to be affected with great and sudden changes from heat to cold alternately. After the high duties were laid on, he gave up the distilling business and took himself to farming, which is his present employment. He was married and had several children; his wife died about a year and a half since, and within this last six months he has married a young woman. When I considered this patient's former course of life, and the state of matrimony which he had lately entered into, I concluded that the cause of the thirst was debility. I once saw a case of a gouty patient who was affected with a very violent thirst instead of a paroxysm of the gout; it yielded to nearly the same treatment as was employed for this patient. With the intention of removing the debility, and restoring the system to its former degree of strength, I had recourse to tonics. Considering the cortex cortex to be among the chief of this kind of medicines, I prescribed it as follows:

Take of fine powder of Peruvian bark, one ounce; sulphat of zinc, half a drachm; mix them accurately together, and divide into sixteen doses; one to be taken four times every day in a glass full of wine.

I ordered porter for his common drink, and a nourishing diet. After using the bark for four days he complained of costiveness; I ordered him one ounce of castor oil, which operated very well. To palliate the uneasy thirst, I prescribed the tamarind decoction. After he had used these medicines for eight days he found his thirst much diminished. He is now taking an opiate every night, and fifteen drops of the acid elixir of vitriol in a glass full of water, three or four times every day; and a dose of castor oil every seven days, which, he says, relieves the uneasiness and hardness of his belly very much. By persisting in this course for four weeks his thirst was almost gone. To perfect the cure, I prescribed for him a journey into the country, that he might have the advantage of a change of air, and drink Chalybeate water; but he could not get time spared for the purpose, and, as he was better, he continued at his usual employment at home.

*Aberdeen, Aug.* 3, 1800. TUAAM PEAAL.

---

